# BioPrev-C – development and validation of a contemporary prostate cancer risk calculator

**DOI:** 10.3389/fonc.2024.1343999

**Published:** 2024-02-21

**Authors:** Thomas Hermanns, Marian S. Wettstein, Basil Kaufmann, Noémie Lautenbach, Ernest Kaufmann, Karim Saba, Florian A. Schmid, Andreas M. Hötker, Michael Müntener, Martin Umbehr, Cédric Poyet

**Affiliations:** ^1^Department of Urology, University Hospital Zürich, University of Zürich, Zürich, Switzerland; ^2^Institute of Diagnostic and Interventional Radiology, University Hospital Zurich, University of Zurich, Zurich, Switzerland; ^3^Department of Urology, Stadtspital Triemli, Zürich, Switzerland

**Keywords:** prostate cancer, biopsy, prostate-specific antigen, nomograms, decision aids

## Abstract

**Objectives:**

To develop a novel biopsy prostate cancer (PCa) prevention calculator (BioPrev-C) using data from a prospective cohort all undergoing mpMRI targeted and transperineal template saturation biopsy.

**Materials and methods:**

Data of all men who underwent prostate biopsy in our academic tertiary care center between 11/2016 and 10/2019 was prospectively collected. We developed a clinical prediction model for the detection of high-grade PCa (Gleason score ≥7) based on a multivariable logistic regression model incorporating age, PSA, prostate volume, digital rectal examination, family history, previous negative biopsy, 5-alpha-reductase inhibitor use and MRI PI-RADS score. BioPrev-C performance was externally validated in another prospective Swiss cohort and compared with two other PCa risk-calculators (SWOP-RC and PBCG-RC).

**Results:**

Of 391 men in the development cohort, 157 (40.2%) were diagnosed with high-grade PCa. Validation of the BioPrev C revealed good discrimination with an area under the curve for high-grade PCa of 0.88 (95% Confidence Interval 0.82-0.93), which was higher compared to the other two risk calculators (0.71 for PBCG and 0.84 for SWOP). The BioPrev-C revealed good calibration in the low-risk range (0 - 0.25) and moderate overestimation in the intermediate risk range (0.25 - 0.75). The PBCG-RC showed good calibration and the SWOP-RC constant underestimation of high-grade PCa over the whole prediction range. Decision curve analyses revealed a clinical net benefit for the BioPrev-C at a clinical meaningful threshold probability range (≥4%), whereas PBCG and SWOP calculators only showed clinical net benefit above a 30% threshold probability.

**Conclusion:**

BiopPrev-C is a novel contemporary risk calculator for the prediction of high-grade PCa. External validation of the BioPrev-C revealed relevant clinical benefit, which was superior compared to other well-known risk calculators. The BioPrev-C has the potential to significantly and safely reduce the number of men who should undergo a prostate biopsy.

## Introduction

Several multivariable risk-assessment tools for better prostate cancer (PCa) risk prediction have been developed in the past (1–3). To reduce unnecessary biopsies and overdiagnosis of low-grade PCa, multivariable risk calculators (RCs) are nowadays recommended by several clinical guidelines (4, 5).

Several studies have shown that RC performance varies when tested in different cohorts (6–10). A RC developed for a specific region might have advantages over RCs developed using cohorts from other geographical regions with different ethnic compositions. Most of the RCs used in daily clinical practice were developed on older biopsy cohorts without information from mpMRI and without the use of targeted biopsies. In recent years however, biopsy practice has widely been changed due to the use of mpMRI and novel biopsy strategies. In our Institution, mpMRI fusion targeting biopsy with additional systematic saturation biopsies has been the usual biopsy strategy in the last years for most men with suspected high-grade PCa due to the increased demand of focal therapy (11).

Here we present the development and validation of a novel RC for PCa detection. The RC was developed on a contemporary cohort of men all undergoing transperineal saturation biopsy including MRI targeting biopsy. No RC developed on saturation biopsy protocol is available so far. This specific aspect makes this RC unique as it potentially lowers the probability that high-grade PCa is missed on biopsy. We specifically aimed to study whether a local developed RC outperforms well known RCs when used in an independent cohort in the same geographic area.

## Materials and methods

For the development of this RC we used prospectively collected data from prostate biopsy database of the Department of Urology of the University Hospital Zurich, Switzerland. All men who underwent prostate biopsy for either an elevated PSA or positive digital rectal examination (DRE) without any history of PCa in our department between 02/2016 and 07/2019 were consecutively included prior biopsy. The recommendation for a biopsy was based on individual recommendation of the treating urologist and not part of the study protocol. Exclusion criteria were patients who had undergone transrectal confirmatory biopsy because of strong suspicion of locally advanced and/or metastatic disease or patients who had not provided informed consent. This cohort is part of the Prostate Biopsy Collaborative Group (PBCG), a large North American and European multicenter study aiming to provide a large prospective multicenter-database of prostate biopsy outcome (12–14).

Before biopsy, all men underwent mpMRI according to PI-RADS guidelines (15), including high-resolution T2-weighted, diffusion-weighted and dynamic contrast-enhanced sequences, acquired on a 3 Tesla MAGNETOM Skyra MRI system (Siemens, Erlangen, Germany). All mpMRIs were evaluated by board-certified radiologists and were reported using the PI-RADS (Prostate Imaging Reporting and Data System) Scoring System, version 2.0 (15).

Prostate biopsies (MRI-targeted fusion and saturation) were done as outpatient procedures under general anesthesia as previously described (11). The BiopSee^®^ MRI/TRUS fusion biopsy system (Medcom) was used for planning and conducting the biopsy. MRI-fusion targeted biopsies (2-3 additional biopsies) were only taken when the mpMRI showed a lesion with a PI-RADS score ≥3. Histopathology was evaluated by a specialized uro-pathologist of our hospital.

A second biopsy cohort was used for validation. This cohort was prospectively collected from 2018 to 2021 at the Triemli Municipal Hospital in Zurich, Switzerland (Triemli cohort), another collaboration partner of the PBCG. In the Triemli cohort all men also underwent pre-biopsy mpMRI with comparable sequences using a 3 Tesla Discovery MR750 MRI system (GE Healthcare, Chicago, United States) before biopsy. All mpMRI were evaluated by board certified radiologists and were reported using the PI-RADS System.

Prostate biopsies were performed as an outpatient procedure usually under local anesthesia and using a transrectal approach using the ARTEMIS system (16). All patients received an individually volumetric-optimized core systematic biopsy. Additionally, targeted fusion biopsies were done in MRI lesions with a PI-RADS score ≥3. Histopathology was evaluated by a specialized uro-pathologist.

Both biopsy outcome studies were approved by the local ethics committee (KEK Nr. 2016-00075 and Amendment PB_2016-00075). All participants of the study provided written informed consent.

The presence/absence of high-grade PCa (Gleason score 7 or greater) was defined as the binary outcome for the RC.

The following parameters were considered as predictors: Age (years), PSA (ng/ml), prostate volume (ml) (measured on MRI images), BMI (kg/m^2^) were investigated as continuous predictors, whereas positive family history, prior negative biopsy, use of 5ARI were used as binary predictors. Finally, two more parameters were categorical predictors: DRE (normal, abnormal, missing) and PIRADS score.

All candidate predictors were investigated by analysis of variance (ANOVA). Continuous predictors with a skewed distribution were truncated (1%) before incorporation. After univariable exploration of all predictors, a limited number multivariable candidate models were investigated. Model building was guided by clinical reasoning and weighing the gain in Chi-squared (χ2) against an additional degree of freedom. With regards to continuous predictors, we further explored the benefit of using restricted cubic splines (3 knots) to account for potential non-linearity. The final model underwent heuristic shrinkage of its coefficients (shrinkage factor: (likelihood ratio – degrees of freedom)/likelihood ratio).

The developed RC was externally validated using the Triemli cohort. Furthermore the RC was benchmarked against two well-known RCs (i.e. PBCG RC, SWOP RC.) The PBCG RC (https://riskcalc.org/PBCG/) is based on multiple heterogeneous cohorts (3) while the SWOP RC (https://www.prostatecancer-riskcalculator.com) is based on the ERSPC Rotterdam cohort for systematic screening (17, 18). Both online available RCs used the same binary outcome (the presence/absence of high-grade PCa) and were thus directly comparable to the BioPrev-C.

Calibration and discrimination of the new RC and the known RCs were performed as previously described (9, 19). Calibration was analyzed graphically using calibration plots and calibration slope. Decision curve analyses (DCAs) for the prediction of high-grade PCa were performed as previously described to assess the net benefit of the RC according to different threshold probabilities at which one would consider performing a biopsy (19, 20). Discrimination was evaluated using receiver-operation characteristic (ROC) curves and the area under the curve (AUC) with corresponding 95% confidence intervals (CI). AUCs were compared using the DeLong Test.

Statistical analyses were performed using R version 3.1.3 (R Foundation for Statistical Computing, Vienna, A) with packages for the calibration plots (21) and DCAs (http://www.decisioncurveanalysis.org) (20).

## Results

### Descriptive analysis of the biopsy cohorts

**Figure 1:**
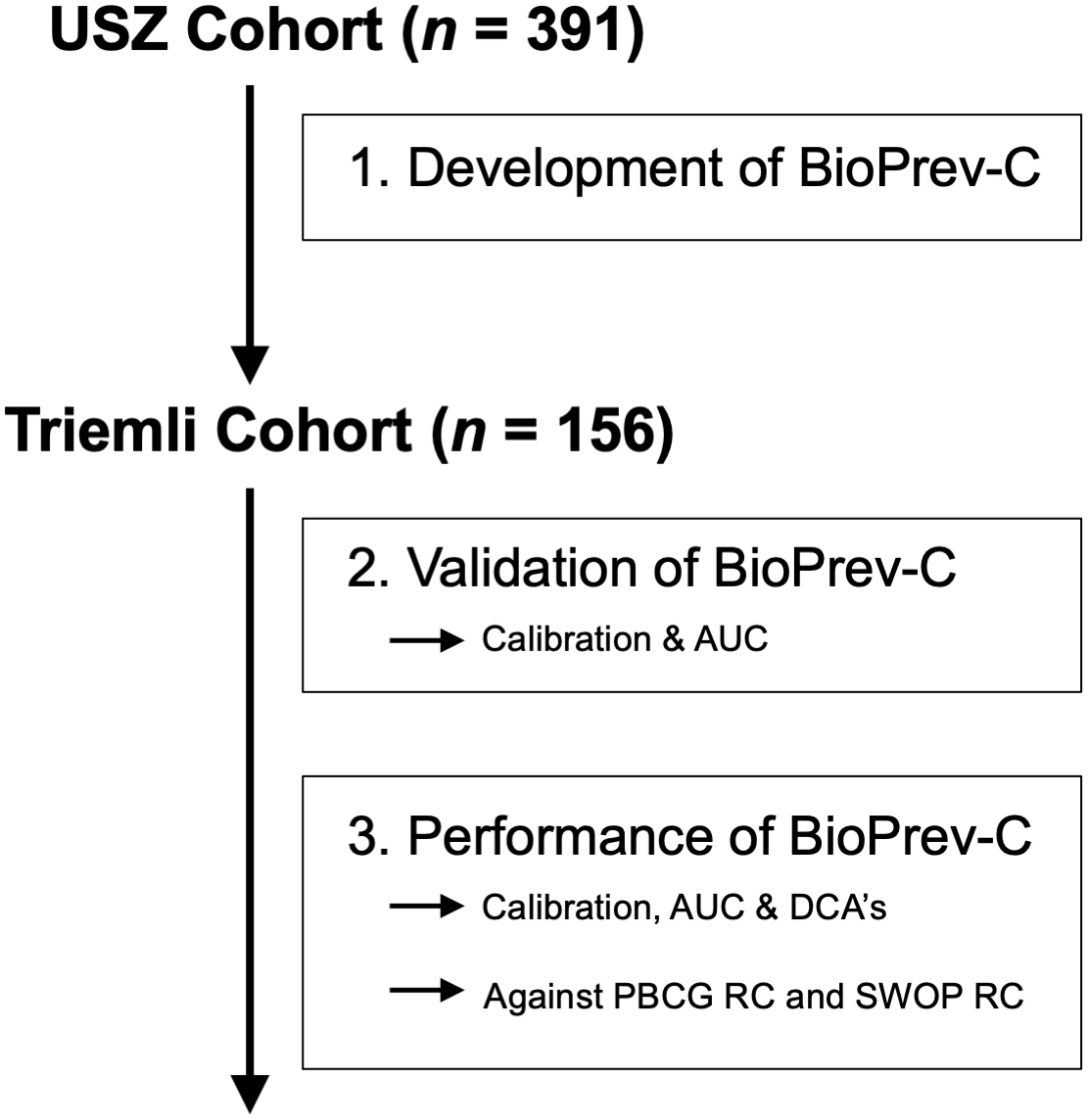
Study flow chart.

A total of 391 men (USZ cohort) were used for the development of the RC. Next, the RC was validated on 156 men from the Triemli cohort. A study flow chart is depicted in **Figure 1**. The baseline characteristics and biopsy results of both cohorts are summarized in **Table 1**. High-grade PCa was found in 157 (40.2%) of all men in the USZ cohort, and in 72 (46.2%) men in the Triemli cohort.

**Table 1:** Baseline characteristics and biopsy results of all eligible men from the development cohort (USZ) and the validation cohort (Triemli).

Variable	Categorization	USZ	Triemli	p-value
Number of patients (n)		391 (100)	156 (100)	
Age (years)		64 (58 - 69)	66 (60.8 - 70)	0.03
PSA (ng/ml)		6.74 (4.80 - 10.10)	7.70 (5.40 - 11.10)	0.04
Prostate volume (mL)		50 (36 - 67)	48 (36 - 60)	0.07
Digital rectal examination				**<0.01**
Abnormal		61 (15.6)	53 (34.0)	
Normal		303 (77.5)	103 (66.0)	
Missing		27 (6.9)	0 (0)	
Family History for PCa				**<0.01**
Yes		79 (20.2)	15 (9.6)	
No		312 (79.8)	141 (90.4)	
Previous negative biopsy				**<0.01**
Yes		114 (29.2)	26 (16.7)	
No		277 (70.8)	130 (83.3)	
5-aplha-reductase inhibitor				0.04
Yes		27 (6.9)	3 (1.9)	
No		364 (93.1)	153 (98.1)	
MRI PIRADS score				**<0.01**
1		94 (24.0)	0 (0)	
2		30 (7.7)	14 (9.0)	
3		87 (22.3)	47 (30.1)	
4		122 (31.2)	37 (23.7)	
5		58 (14.8)	58 (37.2)	
Biopsy outcome				
negative		192 (49.1)	67 (42.9)	
positive		199 (50.9)	89 (57.1)	
	low-grade PCa	42 (10.7)	17 (10.9)	
	high-grade PCa	157 (40.2)	72 (46.2)	
	⇢ GS 7a	57 (14.6)	24 (15.4)	
	⇢ GS 7b	44 (11.3)	25 (16.0)	
	⇢ GS 8	42 (10.7)	13 (8.4)	
	⇢ GS 9&10	14 (3.6)	10 (6.4)	

Data presented as median (Interquartile range) or number (%).

USZ, University Hospital of Zurich, Triemli, City Hospital Triemli of Zurich.

PSA, prostate-specific antigen; PCa, Prostate cancer; PIRADS, Prostate Imaging Reporting and Data.

System, low-grade PCa, PCa with a Gleason Score of 6, high-grade PCa, PCa with a Gleason Score.

7 or higher, GS, Gleason Score.

bold if p-value <0.01.

In the validation cohort more abnormal DRE findings (34% vs. 15%), less previous negative prostate biopsies (16.7 vs. 29.2%) and more PIRADS-5 lesions (37.2% vs. 14.8%) were found compared to the USZ cohort (all <p0.01) (**Table 1**).

### Development of the RC

The continuous candidate predictors PSA, prostate volume, and BMI were truncated due to a skewed distribution (1%). All of the continuous candidate predictors except BMI demonstrated a significant association with the outcome, and, thus, were retained for further evaluation by restricted cubic-splines. The utilization of restricted cubic splines led to an increase in χ2 for all selected continuous variables (age: 13.5 to 19.5; PSA: 4.5 to 4.6; prostate volume: 21.1 to 33.0). However, the increase in χ2 for PSA was not considered worth the additional degrees of freedom. Hence, we decided for a linear incorporation. All binary candidate predictors demonstrated a statistically significant association with the outcome and were considered for the multivariable model. DRE operationalized as a three-level variable (abnormal, normal, missing) was clearly more informative than binary variable (abnormal, normal/missing) and therefore incorporate as a three level variable. The univariable exploration of different forms of operationalization of the candidate predictor PIRADS score was indifferent. As a result, we decided to investigate different forms of operationalization (5 levels [5 versus 4 versus 3 versus 2 versus 1], 4 levels [5 versus 4 versus 3 versus 2/1], 3 levels [5 versus 4 versus 3/2/1]) in the multivariable model. Model simplification (5-level to 4-level to 3-level operationalization) did not lead to a relevant decrease in χ2 (130.8 to 129.6 to 128.6). Hence, the final model involved age in years (restricted cubic spline with 3 knots), PSA in ng/ml, prostate volume in ml (restricted cubic spline with 3 knots), family history (positive versus negative), prior negative biopsy (yes versus no), DRE (abnormal versus missing versus normal) and mpMRI PIRADS (5 versus 4 versus 3/2/1). The univariable and multivariable analyses of all included predictors used for RC development is shown in **Table 2**.

**Table 2:** Univariable and multivariable analyses of all included predictors used for risk calculator development.

Variable	Univariable analysis				Multivariable analysis			
	95% CI				95% CI			
	OR	lower CI level	upper CI level	p-value	OR	lower CI level	upper CI level	p-value
Age	0.94	0.91	0.97	**<0.001**	0.93	0.89	0.97	**<0.001**
PSA	0.96	0.93	0.99	0.004	0.92	0.88	0.97	**<0.002**
Prostate volume^1^	1.05	1.03	1.08	**<0.001**	1.05	1.02	1.08	**0.001**
Prostate volume^2^	0.96	0.93	1.00	0.04	0.99	0.94	1.03	0.59
DRE								
unsuspicious	Ref	Ref	Ref	Ref	Ref	Ref	Ref	Ref
suspicious	0.22	0.12	0.40	**<0.001**	0.53	0.25	1.14	0.11
missing	0.39	0.18	0.87	0.02	0.28	0.10	0.79	0.02
Positive family history	0.45	0.27	0.74	**0.002**	0.56	0.3	1.07	0.08
Prior negative biopsy	1.78	1.12	2.82	0.02	1.74	0.89	3.38	0.1
5-ARI	3.15	1.17	8.52	0.02	2.11	0.61	7.31	0.24
mpMRI results								
PIRADS 1 or 2	Ref	Ref	Ref	Ref	Ref	Ref	Ref	Ref
PIRADS 3	0.5	0.25	1.00	0.05	0.44	0.2	0.94	0.04
PIRADS 4	0.14	0.08	0.25	**<0.001**	0.14	0.07	0.28	**<0.001**
PIRADS 5	0.05	0.02	0.11	**<0.001**	0.06	0.02	0.15	**<0.001**

^1^term 1 of restricted cubic spline with knots at 28, 50 and 97 ml.

^2^term 1 of restricted cubic spline with knots at 28, 50 and 97 ml.

5-ARI, 5-alpha reductase inhibitor; CI, Confidence Intervall; DRE, Digital rectal examination; mpMRI, multiparametric MRI; OR, Odds ratio; PIRADS, Prostate Imaging Reporting and Data System; PSA, prostate-specific antigen.

bold if p-value <0.01.

### External validation

**Figure 2:**
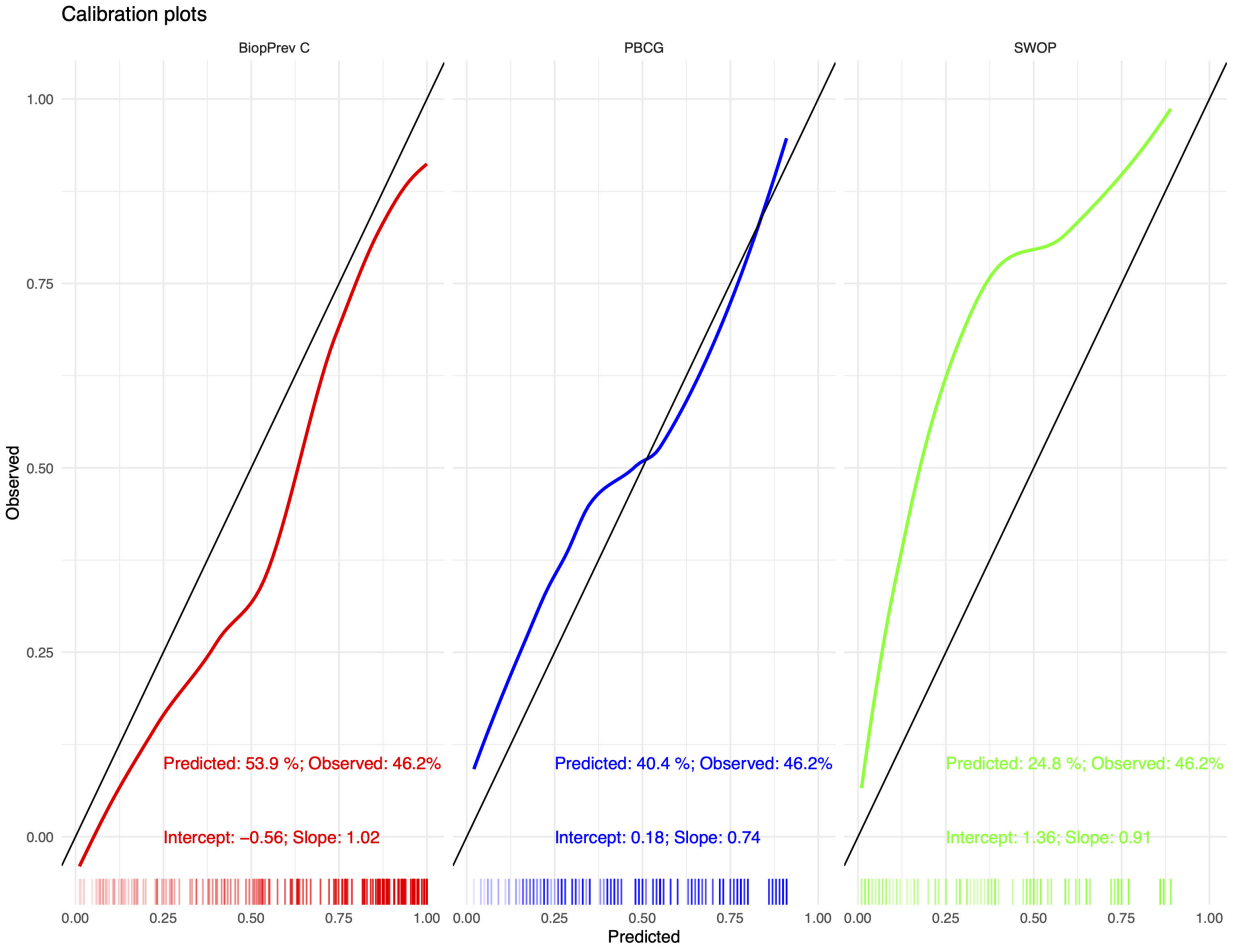
Calibration plots for the BiopPrev-C (Right), the PBCG RC (middle) and the SWOP RC (left) predicting high-grade prostate cancer. The x-axis shows predicted probabilities by the models and the y-axis shows the observed values.

**Figure 3:**
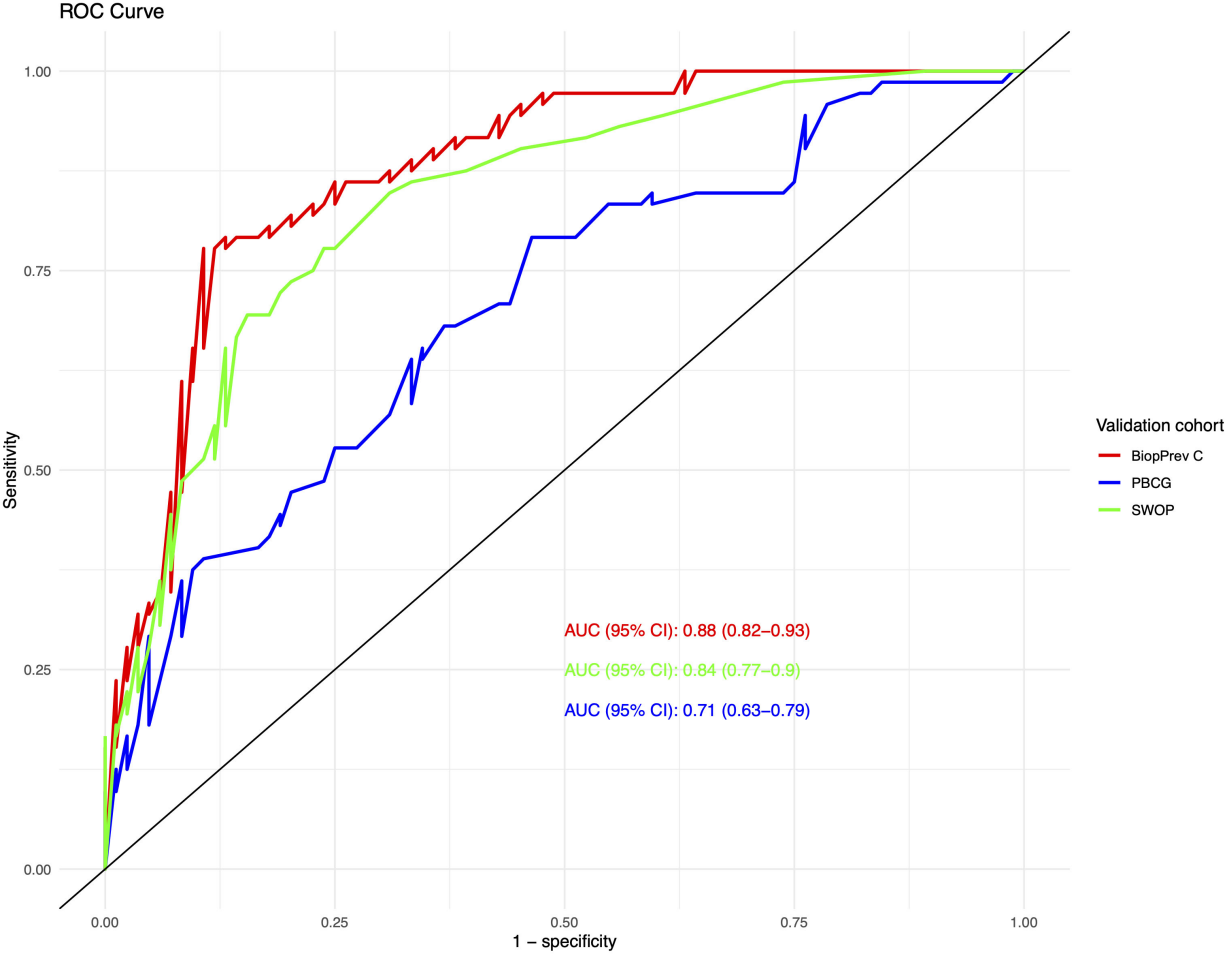
Discrimination of the three risk calculators using a ROC analysis with corresponding AUC values for discriminating a biopsy harbouring high-grade prostate cancer.

**Figure 4:**
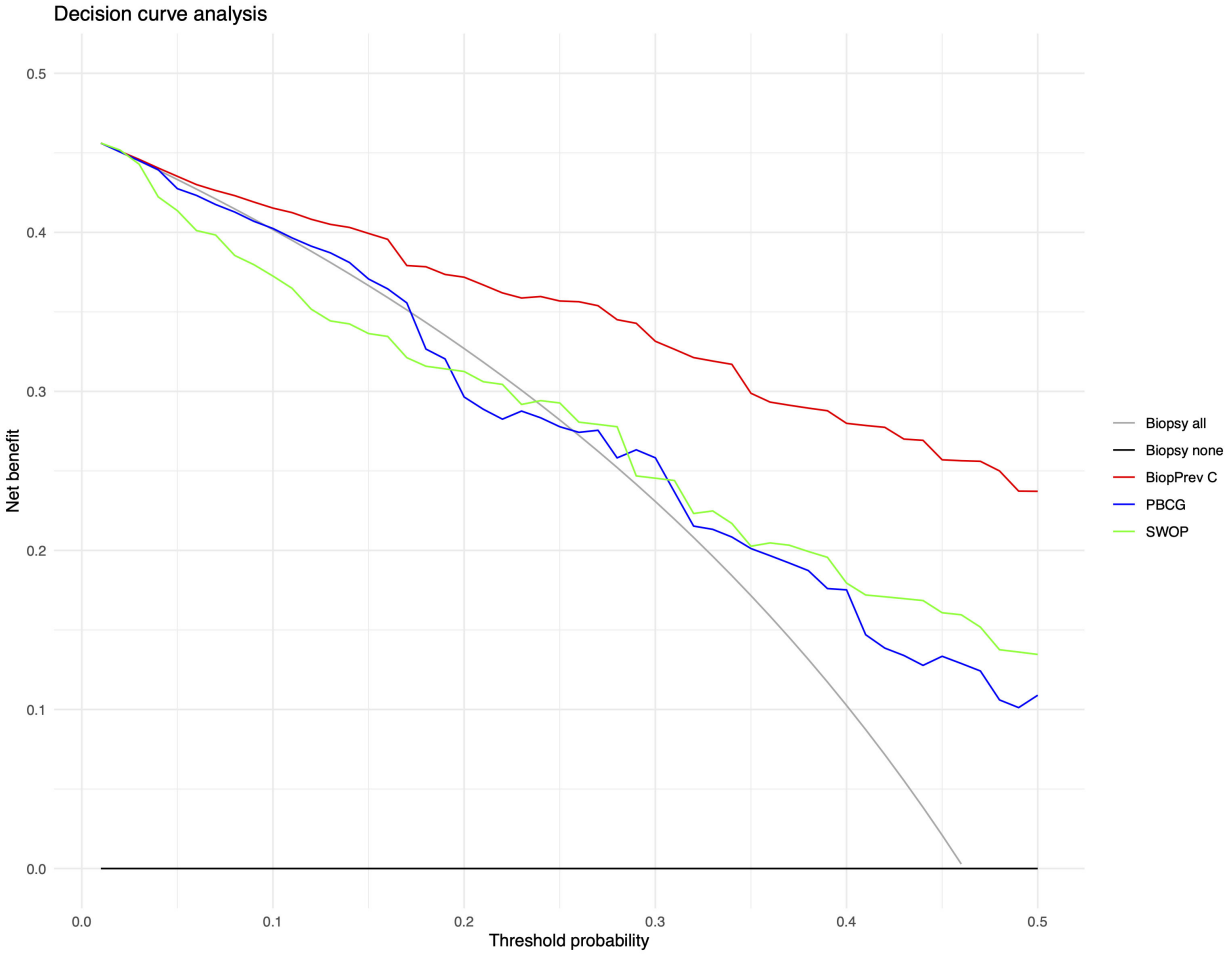
Decision curve analysis for the prediction of high-grade prostate cancer upon biopsy using the either BiopPrev-C, the PBCG RC or the SWOP RC. Decision cures examine the theoretical relationship between the threshold probability of prostate cancer biopsy outcome and the releative value of false-positive and false-negative results to determine the value (net benefit) of a predictive model. The horizontal line along the x-axis assumes that no patient will have prostate cancer (i.e no patient should undergo a prostate biopsy) whereas the solid gray line assumes that all patients will have high-grade prostate cancer (i.e., all patients will need to undergo prostate biopsy).

The developed BioPrev-C was next validated with an independent, external biopsy cohort (Triemli cohort). Calibration plots showed good calibration in low-risk range (0 - 0.25) and moderate overestimation in the intermediate risk range (0.25-0.75) for the BiopPrev-C (**Figure 2**, left). Analyses for the discriminative ability to detect high-grade PCa showed an AUC of 0.88 (95% Confidence Interval (CI) 0.82 - 0.93) (**Figure 3**). DCAs revealed a clinical net benefit for the BiopPrev-C in the threshold probability range between 4% and 50% (**Figure 4**).

Next BioPrev-C performance was benchmarked against the PBCG RC and the SWOP RC. All Variables used for our own RC and for the PBCG and the SWOP RC are summarized in **Table 3**. The PBCG RC showed good calibration over the whole prediction range (**Figure 2**, middle). In contrast, the SWOP calculator showed constant underestimation of high-grade PCa over the whole prediction range (**Figure 2**, right). Calibration-in-the-large showed a predicted rate of 53.9% for the BioPrev-C, 40.4% for the PBCG RC and 24.8% to an actual detection rate of high-grade PCa of 46.2% (**Figure 2**). The AUC of both the PBCG RC (0.71, 95% CI 0.63 - 0.79) and the SWOP RC (0.84, 95% CI 0.77 – 0.90) were significantly lower compared to BioPrev-C (0.88, 95% CI 0.82 – 0.93; BioPrev-C versus PBCG RC: p < 0.001; BioPrev-C versus SWOP: p = 0.02) (**Figure 3**).Finally DCA’s of the other RCs showed inferior clinical net benefit in comparison to the BiopPrev-C. DCA’s revealed only a benefit above the 30% threshold probability for both RCs (PBCG and SWOP RC) (**Figure 4**).

**Table 3:** Characteristics of the three RCs used for validation purposes.

	BiopPrev-C	PBCG RC	SWOP RC
Variables			
Age	x	x	-
PSA	x	x	x
Digital rectal examination	x	x	x
Prostate volume	x	-	x
Prior negative biopsy	x	x	x
First-degree family history	x	x	-
5-aplha-reductase inhibitor	x	-	-
MRI Classification System	x	-	x
**Prediction**	high-grade PCa	high-grade PCa	high-grade PCa

BiopPrev C, Biopsy Prevention Calculator;

PBCG RC, Prostate Biopsy Collaborative Group Risk Calculator.

SWOP RC, Prostate Cancer Research Foundation Risk Calculator.

PSA, prostate-specific antigen; PCa, Prostate cancer; MRI, Magnetic Resonance Imaging.

high-grade PCa, PCa with a Gleason Score of 7 or higher.

## Discussion

Multivariable risk prediction for PCa has been shown to result in a better prediction of high-grade PCa before biopsy. The use of multivariable RC’s instead of PSA values alone should be favoured in predicting the outcome of prostate biopsies” and is thus also recommended by EAU guidelines. In recent years, the use of mp MRI and novel biopsy techniques have changed the way PCa is detected. We report the performance of a newly developed BioPrev-C for the prediction of high-grade PCa risk prediction developed on a prospective biopsy cohort of 391men who all underwent mpMRI and transperineal saturation template prostate biopsy with additional targeted biopsy in case of a PI-RADS lesion ≥ 3.

We validated the BioPrev-C on an independent external biopsy cohort and could show that the BiopPrev-C showed good discrimination (AUC 0.88) and calibration (particularly the low-risk range). Furthermore, the BiopPrev-C revealed a clinical net benefit in DCAs over a large probability range between 4 and 50% and outperformed two other well-known RCs (SWOP-RC and PBCG-RC) in our validation study.

In comparison to other RCs for PCa risk prediction, the BiopPrev-C is based on a contemporary development cohort, in which all men underwent an mpMRI before biopsy. Furthermore, the development cohort is characterized by its extensive saturation (around 40 biopsy cores) and targeted biopsy protocol for all men. This aspect makes the BiopPrev-C unique as it lowers the probability that high-grade PCa is missed on biopsy and that it is present in the group with low-grade PCa or no PCa (22, 23). The relative high certainty of true absence of high-grade PCa in case of a negative biopsy (saturation biopsy protocol) is an important and unique aspect of this RC in comparison to other RCs developed on lower number of biopsies.

We found that the performance of the other two well-known RCs (SWOP-RC and PBCG-RC) were less optimal compared to the BiopPrev-C. Less but not different predictors were used by PBCG and SWOP-RC in comparison to our RC. While the PBCG-RC does not use MRI and prostate volume information, SWOP RC does not use age and PCa family history. We assume that incorporation of more predictors as well as similarity of development and validation cohort might have led to the superior performance of BioPrev-C compared to the other two RC’s. However, we cannot prove this with the available data. A lot of other different reasons have been mentioned in the past for the limitations of a one-size-fits-all RC. Different biopsy strategies or differences in patient populations between a development and a tested cohort (3, 6, 10, 24, 25) are some of these potential limitations. In the current study development and validation cohorts are very similar in terms of biopsy strategy. All men in both cohorts underwent mpMRI before biopsy and both systematic and targeted biopsy. Furthermore, the validation cohort is in close vicinity to the development cohort, what potentially makes the cohorts also more similar with regards to other aspects (Ethnicity, Referral strategies). In summary, our study shows that a local developed RC showed better performance on a local validation cohort then well-known international RCs. However, in a previous work (19) we were not able to show superiority of a local developed PCa RC when applied locally. Though, it is important to note, that the two studies have important differences: In the current study development and validation cohorts are very similar in terms of biopsy strategy. In contrast, important differences between development and validation cohort have been noted (different biopsy strategy, population based mass screening vs. individualized screening) in the previous study. It seems that cohort differences as mentioned above are more important compared to a close regional vicinity between development and validation cohort.

We conclude that our BioPrev-C is of benefit when applied in geographical Even though our actual results of the current Bioprev-C are encouraging, further validation studies are needed especially when not applied in middle Europe or when a different biopsy strategy is used.

A strength of our study is that both cohorts (development and validation) were prospectively recruited and thus variables were all complete with the exception of a few missing DRE’s within the development cohort. This is of importance, as often relevant clinical data for positive biopsy prediction such as precise family history, 5-ARI use is missing in retrospective cohorts. Furthermore, the saturation and targeted biopsy protocol used in the development cohort makes the presence of high-grade PCa in the group with low-grade PCa or no PCa very unlikely.

Recent research has also focused on adding additional biomarkers for the prediction of high-grade PCa. This includes for example the Prostate Health index (PHI) (26), the Proclarix test (27), 4KScore (28) or more recently the Stockholm3 test (29, 30). However, none of the markers has made it yet into daily clinical practice so far for different reasons (costs, practicability, conflicting results). Our proposed PCa RC is a simple ready to use-tool tool in men undergoing state-of-the art systematic and targeted biopsy. Depending on further research a molecular can be implemented into an existing RC for further improvement. We believe that further validation of the BiopPrev-C on different independent biopsy cohorts would be of scientific value for the future.

## Conclusion

BioPrev-C is a novel contemporary prediction tool for the detection of high-grade PCa. Saturation biopsy protocol and mpMRI were performed in all men in the development cohort and thus true absence of high-grade PCa in case of negative biopsy is very likely. BiopPrev-C revealed a relevant clinical benefit in an external validation cohort which was superior to other well-known RCs. The BioPrev-C has the potential to significantly and safely reduce the number of men who should undergo a prostate biopsy.

## Data availability statement

The raw data supporting the conclusions of this article will be made available by the authors, without undue reservation.

## Ethics statement

The studies involving humans were approved by local ethics committee Zurich (KEK Nr. 2016-00075 and Amendment PB_2016-00075). The studies were conducted in accordance with the local legislation and institutional requirements. The participants provided their written informed consent to participate in this study.

## Author contributions

TH: Data curation, Methodology, Writing – original draft, Writing – review & editing. MW: Methodology, Validation, Writing – review & editing. BK: Data curation, Writing – review & editing. NL: Data curation, Formal Analysis, Writing – review & editing. EK: Data curation, Writing – review & editing. KS: Data curation, Methodology, Writing – review & editing. FS: Writing – review & editing. AH: Writing – review & editing. MM: Data curation, Writing – review & editing. MU: Data curation, Resources, Writing – review & editing. CP: Conceptualization, Methodology, Supervision, Writing – original draft, Writing – review & editing.

## References

[1] Bandala-Jacques A Castellanos Esquivel KD Perez-Hurtado F Hernandez-Silva C Reynoso-Noveron N. Prostate cancer risk calculators for healthy populations: systematic review. JMIR Cancer (2021) 7:e30430. doi: 10.2196/30430 34477564 PMC8449298

[2] Cavadas V Osorio L Sabell F Teves F Branco F Silva-Ramos M. Prostate cancer prevention trial and European randomized study of screening for prostate cancer risk calculators: a performance comparison in a contemporary screened cohort. Eur Urol (2010) 58:551–8. doi: 10.1016/j.eururo.2010.06.023 20580483

[3] Ankerst DP Straubinger J Selig K Guerrios L De Hoedt A Hernandez J. A contemporary prostate biopsy risk calculator based on multiple heterogeneous cohorts. Eur Urol (2018) 74:197–203. doi: 10.1016/j.eururo.2018.05.003 29778349 PMC6082177

[4] Mottet N van den Bergh RCN Van den Broeck T Cumberbatch MG De Santis M. EAU-EANM-ESTRO-ESUR-ISUP-SIOG guidelines on prostate cancer (2021). doi: 10.1016/j.eururo.2020.09.042 33039206

[5] Van Poppel H Roobol MJ Chapple CR Catto JWF N’Dow J Sonksen J. Prostate-specific antigen testing as part of a risk-adapted early detection strategy for prostate cancer: European association of urology position and recommendations for 2021. Eur Urol (2021) 80:703–11. doi: 10.1016/j.eururo.2021.07.024 34407909

[6] Ankerst DP Boeck A Freedland SJ Jones JS Cronin AM Roobol MJ. Evaluating the Prostate Cancer Prevention Trial High Grade Prostate Cancer Risk Calculator in 10 international biopsy cohorts: results from the Prostate Biopsy Collaborative Group. World J Urol (2014) 32:185–91. doi: 10.1007/s00345-012-0869-2 PMC3702682 22527674

[7] Lundon DJ Kelly BD Foley R Loeb S Fitzpatrick JM Watson RW. Prostate cancer risk assessment tools in an unscreened population. World J Urol (2014) 33(6):827–32. doi: 10.1007/s00345-014-1365-7 25091862

[8] Trottier G Roobol MJ Lawrentschuk N Bostrom PJ Fernandes KA Finelli A. Comparison of risk calculators from the Prostate Cancer Prevention Trial and the European Randomized Study of Screening for Prostate Cancer in a contemporary Canadian cohort. BJU Int (2011) 108:E237–44. doi: 10.1111/j.1464-410X.2011.10207.x 21507190

[9] Poyet C Nieboer D Bhindi B Kulkarni GS Wiederkehr C Wettstein MS. Prostate cancer risk prediction using the novel versions of the ERSPC and PCPT risk calculators: Independent validation and comparison in a contemporary European cohort. BJU Int (2015) 117(3):401–8. doi: 10.1111/bju.13314 10.1111/bju.13314 26332503

[10] Saba K Wettstein MS Lieger L Hotker AM Donati OF Moch H. External validation and comparison of prostate cancer risk calculators incorporating multiparametric magnetic resonance imaging for prediction of clinically significant prostate cancer. J Urol (2020) 203:719–26. doi: 10.1097/JU.0000000000000622 31651228

[11] Kaufmann B Saba K Schmidli TS Stutz S Bissig L Britschgi AJ. Prostate cancer detection rate in men undergoing transperineal template-guided saturation and targeted prostate biopsy. Prostate (2022) 82:388–96. doi: 10.1002/pros.24286 PMC9299705 34914121

[12] Vickers AJ Cronin AM Roobol MJ Hugosson J Jones JS Kattan MW. The relationship between prostate-specific antigen and prostate cancer risk: the Prostate Biopsy Collaborative Group. Clin Cancer Res (2010) 16:4374–81. doi: 10.1158/1078-0432.CCR-10-1328 PMC2937360 20736330

[13] Clements MB Vertosick EA Guerrios-Rivera L De Hoedt AM Hernandez J Liss MA. Defining the impact of family history on detection of high-grade prostate cancer in a large multi-institutional cohort. Eur Urol (2021) 82(2):163–9. doi: 10.1016/j.eururo.2021.12.011 PMC9243191 34980493

[14] Tolksdorf J Kattan MW Boorjian SA Freedland SJ Saba K Poyet C. Multi-cohort modeling strategies for scalable globally accessible prostate cancer risk tools. BMC Med Res Methodol (2019) 19:191. doi: 10.1186/s12874-019-0839-0 31615451 PMC6792191

[15] Weinreb JC Barentsz JO Choyke PL Cornud F Haider MA Macura KJ. PI-RADS prostate imaging - reporting and data system: 2015, version 2. Eur Urol (2016) 69:16–40. doi: 10.1016/j.eururo.2015.08.052 26427566 PMC6467207

[16] Natarajan S Marks LS Margolis DJ Huang J Macairan ML Lieu P. Clinical application of a 3D ultrasound-guided prostate biopsy system. Urologic Oncol (2011) 29:334–42. doi: 10.1016/j.urolonc.2011.02.014 PMC3432280 21555104

[17] Roobol MJ van Vugt HA Loeb S Zhu X Bul M Bangma CH. Prediction of prostate cancer risk: the role of prostate volume and digital rectal examination in the ERSPC risk calculators. Eur Urol (2012) 61:577–83. doi: 10.1016/j.eururo.2011.11.012 22104592

[18] Alberts AR Roobol MJ Verbeek JFM Schoots IG Chiu PK Osses DF. Prediction of high-grade prostate cancer following multiparametric magnetic resonance imaging: improving the rotterdam European randomized study of screening for prostate cancer risk calculators. Eur Urol (2019) 75:310–8. doi: 10.1016/j.eururo.2018.07.031 30082150

[19] Poyet C Wettstein MS Lundon DJ Bhindi B Kulkarni GS Saba K. External evaluation of a novel prostate cancer risk calculator (ProstateCheck) based on data from the Swiss arm of the ERSPC. J Urol (2016) 196:1402–7. doi: 10.1016/j.juro.2016.05.081 PMC5576554 27188476

[20] Vickers AJ Elkin EB. Decision curve analysis: a novel method for evaluating prediction models. Med decision making: an Int J Soc Med Decision Making (2006) 26:565–74. doi: 10.1177/0272989X06295361 PMC2577036 17099194

[21] Kundu S Aulchenko YS van Duijn CM Janssens AC. PredictABEL: an R package for the assessment of risk prediction models. Eur J Epidemiol (2011) 26:261–4. doi: 10.1007/s10654-011-9567-4 PMC3088798 21431839

[22] Crawford ED Rove KO Barqawi AB Maroni PD Werahera PN Baer CA. Clinical-pathologic correlation between transperineal mapping biopsies of the prostate and three-dimensional reconstruction of prostatectomy specimens. Prostate (2013) 73:778–87. doi: 10.1002/pros.22622 PMC4625901 23169245

[23] Marra G Eldred-Evans D Challacombe B Van Hemelrijck M Polson A Pomplun S. Pathological concordance between prostate biopsies and radical prostatectomy using transperineal sector mapping biopsies: validation and comparison with transrectal biopsies. Urol Int (2017) 99:168–76. doi: 10.1159/000471491 28768264

[24] Nam RK Kattan MW Chin JL Trachtenberg J Singal R Rendon R. Prospective multi-institutional study evaluating the performance of prostate cancer risk calculators. J Clin Oncol (2011) 29:2959–64. doi: 10.1200/JCO.2010.32.6371 21690464

[25] Kattan MW. Factors affecting the accuracy of prediction models limit the comparison of rival prediction models when applied to separate data sets. Eur Urol (2011) 59:566–7. doi: 10.1016/j.eururo.2010.11.039 21156336

[26] Loeb S Shin SS Broyles DL Wei JT Sanda M Klee G. Prostate Health Index improves multivariable risk prediction of aggressive prostate cancer. BJU Int (2017) 120:61–8. doi: 10.1111/bju.13676 PMC5392379 27743489

[27] Gentile F La Civita E Ventura BD Ferro M Bruzzese D Crocetto F. A neural network model combining [-2]proPSA, freePSA, total PSA, cathepsin D, and thrombospondin-1 showed increased accuracy in the identification of clinically significant prostate cancer. Cancers (Basel) (2023) 15. doi: 10.3390/cancers15051355 PMC10000171 36900150

[28] Verbeek JFM Bangma CH Kweldam CF van der Kwast TH Kummerlin IP van Leenders G. Reducing unnecessary biopsies while detecting clinically significant prostate cancer including cribriform growth with the ERSPC Rotterdam risk calculator and 4Kscore. Urologic Oncol (2019) 37:138–44. doi: 10.1016/j.urolonc.2018.11.021 30528698

[29] Nordstrom T Discacciati A Bergman M Clements M Aly M Annerstedt M. Prostate cancer screening using a combination of risk-prediction, MRI, and targeted prostate biopsies (STHLM3-MRI): a prospective, population-based, randomised, open-label, non-inferiority trial. Lancet Oncol (2021) 22:1240–9. doi: 10.1016/S1470-2045(21)00348-X 34391509

[30] Palsdottir T Gronberg H Hilmisson A Eklund M Nordstrom T Vigneswaran HT. External validation of the rotterdam prostate cancer risk calculator and comparison with stockholm3 for prostate cancer diagnosis in a Swedish population-based screening cohort. Eur Urol Focus (2022) 9(3):455–62. doi: 10.1016/j.euf.2022.11.021 36522257

